# Ovarian Yolk Sac Tumour in a Girl − Case Report

**DOI:** 10.34763/devperiodmed.20172102.101103

**Published:** 2017-08-11

**Authors:** Charu Sharma, Hemanshi Shah, Neha Sisodiya Shenoy, Deepa Makhija, Mukta Waghmare

**Affiliations:** 1 Dept of Paediatric Surgery, TNMC & BYL Nair Hospital, Mumbai Central, Mumbai, Maharashtra. India. Pin: 400008

**Keywords:** Yolk sac tumour, ovary, girl

## Abstract

Yolk sac tumours are rare ovarian malignancies accounting for less than 1% of malignant ovarian germ cell tumours. They are mostly seen in adolescents and young women and are usually unilateral making fertility preservation imperative. Raised alpha-feto protein level is the hallmark of this tumour. We describe stage III yolk sac tumour in a girl child.

## Introduction

Yolk sac tumour of the ovary, also known as endodermal sinus tumour, is a rare malignant ovarian germ cell tumour (MOGCT) [1]. It accounts for less than 1% of all ovarian tumours [1, 2]. It is frequently seen in adolescents and young women, which makes it imperative to preserve fertility during management. Adjuvant chemotherapy consisting of Bleomycin-Etoposide-Cisplatin (BEP) regimen has greatly improved the outcomes of these tumours.

## Case Summary

A 9-year-old girl presented with a history of gradual abdominal distention that had started three months before. It was associated with a dull aching pain in the abdomen.

**Fig. 1:**
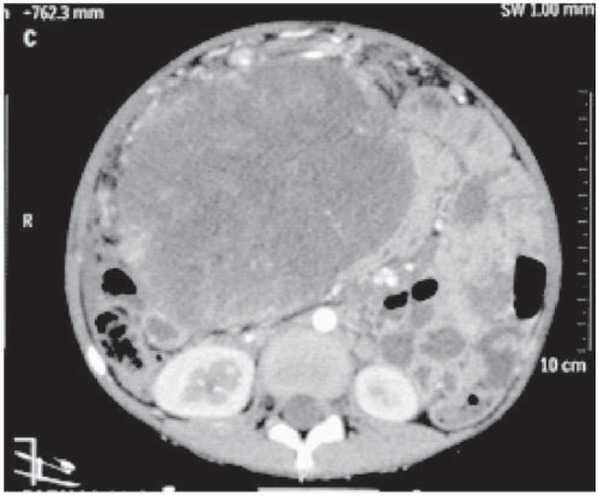
CT scan (axial section) showing the large solid cystic pelvic mass with septations.

**Fig. 2:**
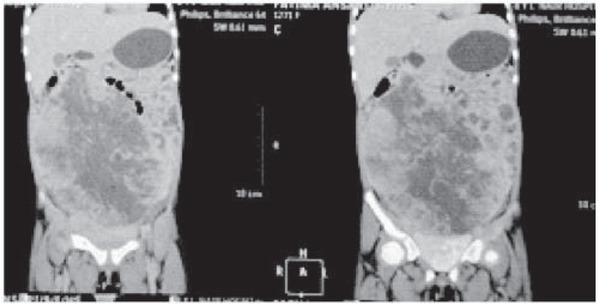
CT scan (Saggital section) showing large solid cystic adnexal/ovarian mass with septations and peritoneal deposits.

The girl was pale with massive abdominal distension and mild respiratory distress. A large 12 cm x 12 cm firm mass arising from the pelvis was palpable and occupied almost all of the abdomen. Blood investigations suggested anaemia. Lactate Dehydrogenase and Alpha-FetoProtein were raised (1143 U/l and more than 1000). Computed Tomography suggested a large solid cystic adnexal/ ovarian mass with septations and pedunculated serosal deposits on sigmoid colon and mild ascites suggesting neoplastic etiology (fig. 1 and 2).

Exploratory laparotomy revealed a 15x13x12cm bosselated mass arising from the left ovary with the omentum and appendix adhering to it, mild haemorrhagic ascites and pedunculated serosal deposits over terminal ileum and lower sigmoid. There were peritoneal deposits between the two iliac vessels and multiple enlarged mesenteric lymph nodes. The right ovary, right fallopian tube and uterus were normal. left salpingo-oophorectomy, omentectomy, appendicectomy with removal of the serosal deposits over the terminal ileum and sigmoid was done. Histopathology suggested yolk sac tumour. The patient recovered uneventfully and is now on BEP (Bleomycin, Etoposide and Cisplatin) chemotherapy.

## Discussion

Ovarian germ cell tumours constitute 15 to 20% of all the ovarian tumours [1]. They originate from the primitive germ cell and gradually differentiate to mimic tissues of either the embryonic origin like ectoderm, endoderm and mesoderm or of the extraembryonic tissues like the yolk sac and trophoblast [3]. The specific type of tumour depends on the degree of differentiation [benson]. According to the scheme characterized by Telium, a germinoma would develop if there is no differentiation; with differentiation, embryonal carcinoma would develop and with extraembryonic differentiation, a yolk sac tumour or a choriocarcinoma [4].

MOGCTs account for 3 to 5% of all the ovarian malignancies and are subdivided into germinomatous and non-germinomatous tumours [2, 3]. Yolk sac tumour and immature teratoma are the commonest type of non-germinomatous MOGCTs [3, 5]. Yolk sac tumour, though rare, is the second commonest histopathological subtype of malignant ovarian germ cell tumours after dysgerminoma [3].

Yolk sac tumour is usually seen in adolescents and young adults, between 18 to 30 years of age [1, 6]. The clinical symptoms include an enlarging pelvic mass which extends to the abdomen and is associated with pain [3]. The tumour is almost always unilateral and the median diameter is 15 to 19cm [3]. Often there is rapid growth with extensive intra-abdominal spread leading to poor prognosis [3]. Other symptoms are vaginal bleeding, fever, ascites or peritonitis secondary to torsion, infection or tumour rupture [2, 3]. Ascites may lead to diffuse abdominal tenderness, decreased bowel sounds and decreased breath sounds at lung bases [3]. Elevated AFP levels is the hallmark of this tumour and rapid decline in serum levels of AFP indicate absence of residual tumour after surgery [3]. The efficiency of chemotherapy is related to the normalization of the AFP levels [3].

Pre-operative diagnosis is difficult, as yolk sac tumours do not have a specific radiological image [3]. These tumours can appear cystic with signs of hypervascularization and areas of haemorrhage [3]. Locoregional extension involving the uterus, pelvic peritoneum, rectum and bladder suggest malignant evolution [3]. Involvement of omentum, abdominal peritoneum and serosal surfaces of the bowel has been reported in 30% of the cases [3]. In advanced stages, retroperitoneal lymph nodes and liver parenchyma are also involved [2, 3]. The diagnosis is histopathological. Histologically the malignant tissue resembles the structure found in early embryonic development – the Schiller duval bodies [3, 7].

Complete surgical excision is the standard management for these tumours [3]. Fertility-sparing surgery is often possible, as the tumours are unilateral [3]. Recently, minimally invasive surgery has been recommended to offer better prognosis [3]. Nishio et al have reported the type of surgical procedure not to be an important prognostic factor for patients with MOGCTs at all clinical stages, thereby indicating conservative and fertility sparing surgery to be appropriate along with adjuvant chemotherapy [8, 9, 10]. Thus, even in patients with bulky metastases, a normal appearing uterus and contralateral ovary can be safely preserved allowing for future fertility [8]. However, it is recommended that patients with bulky disease in the abdomen, pelvis, and retroperitoneum should be surgically cytoreduced to optimal residual disease if possible [8, 11]. The BEP chemotherapeutic regimen has proved to be efficacious in treating MOGCTs since its introduction in the 1980s [8].

However, there are some toxicities of this BEP regimen - hair loss, fatigue, nausea, and myelosuppression [8]. Cisplatin is known to be associated with nerve damage manifesting as peripheral neuropathy or hearing loss [8]. A potentially fatal side effect of this regimen is bleomycininduced pulmonary fibrosis, making it mandatory for patients to have pulmonary function testing before treatment to document baseline function and allow for surveillance of function during therapy [8]. There is concern for secondary malignancy related to etoposide in the form of acute myelogenous leukemia, related to cumulative dose effect [8]. Yet, these toxicities are relatively uncommon and short-lived when they occur due to the short duration of treatment, the standard being only three cycles [8]. Though there is concern of risk of infertility following chemotherapy, the majority of these patients will maintain their ovarian function and fertility, as reported in various studies [3].

Factors related to good prognosis are no ascites at presentation, stage I disease, less than 42 days to AFP normalization, fertility-sparing surgery and a serum AFP half-life less of 10 days [3]. Progressive or recurrent ovarian tumour after treatment with BEP chemotherapy has been reported to be associated with a poor prognosis [3]. As there are no approved chemotherapy schemes in such cases, the possible options include combination of vinblastine, ifosfamide, cisplatin, or paclitaxel, ifosfamide, cisplatin, as adapted from the regime for testicular cancer [3, 12]. However, it has been stated that secondary cyto-reductive surgery could play an important role when tumours are limited and resistant to chemotherapy [3].

Follow-up of these patients includes determining if there is an initially elevated AFP level and repeating it before each cycle of therapy, soon after the end of the treatment and during the 2 years after the end of chemotherapy [3]. An annual pelvic ultrasound is recommended in cases with conservative surgery in order to screen for a contralateral recurrence [3].
